# Rumor Propagation is Amplified by Echo Chambers in Social Media

**DOI:** 10.1038/s41598-019-57272-3

**Published:** 2020-01-15

**Authors:** Daejin Choi, Selin Chun, Hyunchul Oh, Jinyoung Han, Ted “Taekyoung” Kwon

**Affiliations:** 1 Georgia Institute of Technology, School of Interactive Computing, Atlanta, 30332 USA; 2 Seoul National University, Department of Computer Science and Engineering, Seoul, 08826 Republic of Korea; 3 Sungkyunkwan University, Department of Applied Artificial Intelligence, Seoul, 03063 Republic of Korea

**Keywords:** Applied physics, Environmental social sciences

## Abstract

Spreading rumors on the Internet has become increasingly pervasive due to the proliferation of online social media. This paper investigates how rumors are amplified by a group of users who share similar interests or views, dubbed as an *echo chamber*. To this end, we identify and analyze *‘rumor’ echo chambers*, each of which is a group of users who have participated in propagating common rumors. By collecting and analyzing 125 recent rumors from six popular fact-checking sites, and their associated 289,202 tweets/retweets generated by 176,362 users, we find that the rumors that are spread by rumor echo chamber members tend to be more viral and quickly propagated than those that are not spread by echo chamber members. We propose the notion of an *echo chamber network* that represents relations among rumor echo chambers. By identifying the hub rumor echo chambers (in terms of connectivity to other rumor echo chambers) in the echo chamber network, we show that the top 10% of hub rumor echo chambers contribute to propagation of 24% rumors by eliciting more than 36% of retweets, implying that core rumor echo chambers significantly contribute to rumor spreads.

## Introduction

Online media such as social networks, online communities, instant messages, and e-mails have become popular vectors in disseminating news, content, political campaigns, scientific findings, or product advertisements. Due to the nature of such online media that spreads information quickly and widely^1–3^, there have been attempts to disseminate misinformation, false news, or *rumors*, the last of which are circulating stories of uncertain or ungrounded gossips^4,5^. Such rumors usually affect people, society, or economics. For example, the misinformation about Hurricane Sandy on the east coast of the USA brought the storm of rumors and false photos on the Internet^6^. Another well-known widespread rumor is about the Boston Marathon Bombing event. FBI requested for materials related to the suspects (as bombers), but many people reported and propagated a deluge of misinformation or rumors to that event at the moment^7^. A false news about Barack Obama’s injury due to the White House explosion was reported through Twitter by the Associated Press (AP), which brought monetary chaos in stock markets – $130 billion in stock values were wiped out^8^.

The growing importance in understanding rumor spreads has spurred research into analyzing rumor propagation patterns in online media. By analyzing the factors that are related to rumor propagation, researchers have investigated how different types of content^9,10^ or initial propagation patterns^9^ are associated with the rumor propagation. From a user perspective, the relations between rumor propagation patterns and individuals’ interest (or political standpoint)^11,12^, behavioral characteristics^7,13^, or social structures^14^ have been investigated. While these studies have provided valuable insights into understanding rumor spreads and their associated factors, relatively little attention has been paid to how *a group of users* that share similar interests or political views play roles in rumor propagation.

Social media such as Twitter or Facebook allows users with diverse backgrounds (e.g., political viewpoint, race, or gender) to share news, information, or opinions. Despite such a diversity in social media, studies have reported that online users with similar interests tend to be gathered and eventually form a homogeneous cluster, known as *echo chamber*^15^, resulting in assimilation or even amplification of beliefs or memes. Such nature of echo chambers has attracted researchers to investigate the characteristics or social properties of echo chambers^16–20^ revealed in social media, such as selective exposure or political homophily. However, little attention has been paid to how echo chambers play roles in spreading political rumors in social media.

This paper investigates how political rumors spread in social media are amplified in echo chambers in the first place. To this end, we collected (i) 125 recent rumors whose topics are either about US politics or US politicians from six popular fact-checking sites (snopes.com, politifact.com, factcheck.org, hoaxslayer.com, truthorfiction.com, and urbanlegends.about.com) and (ii) 289,202 English-written tweets/retweets that associated with the 125 rumors generated by 176,362 users in Twitter, and (iii) user information (e.g., tweets shown in his/her timeline, number of followers, etc.) of the collected tweets/retweets. The retrieved tweets had been generated from June 2009 to October 2018. Refer to https://daejin-choi.github.io/datasets/19srep-rumors/ for the detailed information of our collected data. Based on the collected data, we start our investigation by identifying a *rumor echo chamber*, defined as a group of users who participate in propagating at least two common rumors. The rationale behind this approach is that, users may have similar interest if they are involved in propagating common rumors. By investigating the characteristics of the identified rumor echo chambers, we find that rumor echo chambers tend to reveal the selective exposure and political homophily, well-known distinct properties of political echo chambers reported in the social science literature.

Our analysis on rumor echo chamber members, who share common political views or interests, reveals that they are likely to participate in rumor propagation in early stages and hence elicit others’ participation. We find that rumors that are spread by rumor echo chamber members tend to be more viral and quickly propagated than those that are not spread by echo chamber members.

We finally characterize the relations of echo chambers, by proposing the notion of an *echo chamber network*, whose nodes are echo chambers and an edge between two nodes indicates a set of common users who participate in the two echo chambers. We identify the hub echo chambers (in terms of degrees) in the given echo chamber network, and show that the top 10% of hub echo chambers are responsible for 24% of all the rumor cascades, eliciting more than 36% of retweets, which implies that core rumor echo chambers significantly contribute to rumor spreads in social media.

## Results

### Properties of rumor echo chambers

We first characterize the identified rumor echo chambers from two perspectives: *selective exposure* and *political homophily*, well-known properties of political echo chambers reported in the social science literature^19^.

#### Selective exposure

Selective exposure is a social phenomenon that a user tends to selectively consume information that he/she would like to believe^21^, which is considered as a distinct property of (political) echo chambers^19^. Inspired from this, we measure whether the selective exposure is observed in rumor echo chambers identified in this paper. To this end, we first characterize the political polarity of a user in two ways: (i) following-based and (ii) tweet-based. The following-based political polarity of a user, called as *‘user polarity’*, can be calculated based on the latent space model proposed in^18^, which considers politicians who the given user follows. On the other hand, the tweet-based polarity of a user, called *‘content polarity’*, can be calculated with the polarity scores of URLs used in his/her tweets. That is, we calculate the average polarity score of a user over his/her recent 400 tweets/retweets. Note that we use the domain-based polarity score system of 500 news domains in^22^ for the polarity score of each URL. We ignore the tweets/retweets whose content URLs are not in the domain-based polarity system. The values of user and content polarity are in range of [−2, 2] and [0, 1], respectively, and the lowest and the highest value indicates that the given user is close to the left-wing and the right-wing, respectively.

**Figure 1:**
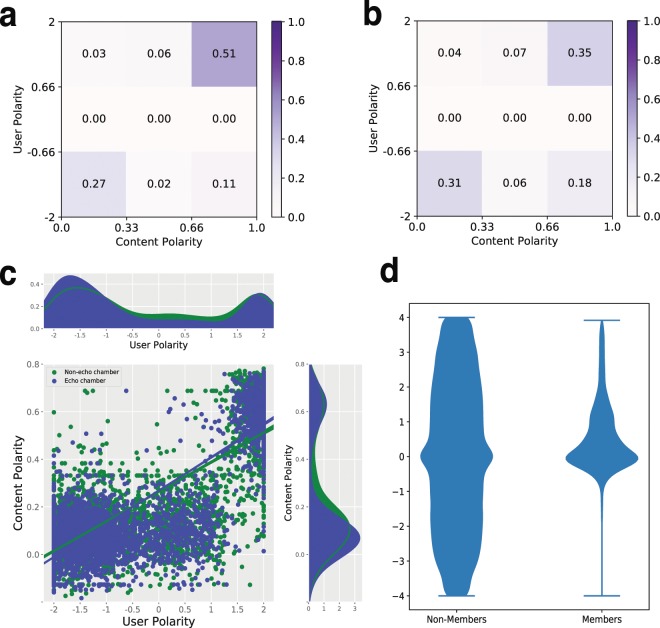
Measuring selective exposure and political homophily in rumor echo chambers. The portions of members (**a**) and non-members (**b**) of rumor echo chambers in terms of content and user polarity are plotted. In both figures, the portions of the users in the left-bottom and right-top cells tend to be higher than others, meaning that users participating in rumor spreads tend to show the selective exposure in general. Comparing (**a**,**b**), the sum of the portions of user in the left-bottom and right-top cells of echo chamber and non-echo chamber are 0.78 and 0.68, respectively, meaning that echo chamber members show the relatively stronger selective exposure than non-members. As a case study, (**c**) shows the user and content polarities of each user for the rumor *“YETI cut ties with the NRA”*. The scatter plot shows the user and content polarities of each user, and one-dimensional plots along the axes reveal the distributions of the user and content polarities. The content polarities of echo chamber members show the stronger bimodal distribution than those of non-members. These results imply that echo chambers tend to exhibit stronger selective exposure. (**d**) shows the distribution of user homogeneity scores of the users who belong to the same echo chamber. For the comparison purpose, we randomly select 100 K pairs of users who do not belong to the same echo chamber, and compute their user homogeneity scores. The result reveals that echo chamber members tend to share similar political views.

Figure 1(a,b) show the portions of members (and non-members) of rumor echo chambers in terms of content and user polarity, respectively. Note that we divide the users into three classes for each polarity; finally, there are 9 user classes based on the user and content polarities. For example, users in the left-bottom cell are likely to be left-wing in terms of both content and user polarities. On the other hand, users in the right-top cell tend to follow (and consume content from) people whose political stances are conservative. As shown in Fig. 1(a,b), the portions of the users in the left-bottom and right-top cells tend to be higher than the ones in other cells, implying that users participating in rumor spreads show the selective exposure in general. The sum of the portion of users in the left-bottom and right-top cells of echo chamber and non-echo chamber are 0.78 and 0.68, respectively, meaning that echo chamber members show the relatively stronger selective exposure than non-members, which is inline with the prior studies^19^.

We further investigate how each user shows the selective exposure in a particular rumor cascade. Figure 1(c) shows the user and content polarities of each user who participates in the cascades of the rumor *“YETI told the NRA the brand no longer wishes to do business with the organization”*. The top and right figures indicate the distributions of user and content polarities, respectively. Note that we divide users into two categories – users participating in echo chambers or not. As shown in Fig. 1(c), users form two clusters in the left-lower and right-upper spaces, meaning that users are likely to consume content similar to their political views. Interestingly, the content polarities of echo chamber members show the stronger bimodal distribution than those of non-members, implying that users in echo chambers tend to exhibit stronger selective exposure.

#### Political homophily

To examine how members in an echo chamber are politically similar, we calculate the *user homogeneity* between two users *i* and *j* in an echo chamber as *σ*_*i*_ = *σ*_*i*_*σ*_*j*_, where *σ*_*i*_ is the user polarity of user *i*. The user homogeneity score is close to −4 if two users are in politically opposite positions, and close to 4 if their political positions are similar.

Figure 1(d) shows the distribution of user homogeneity scores of the users who belong to the same echo chamber. For the comparison purpose, we randomly select 100 K pairs of users who do not belong to the same echo chamber, and compute their user homogeneity scores. As shown in Fig. 1(d), most user homogeneity scores of echo chambers are around 0 and some portion of echo chambers show positive scores while the randomly selected pairs show a broad spectrum in [−4, 4]. In particular, 62.3% of the members in the same echo chamber reveal positive user homogeneity while only half of randomly selected pairs show same political sign. This results indicate that echo chamber members tend to share similar political views.

### Roles of echo chambers in rumor propagation

We next explore how echo chambers play roles in rumor propagation in social media. To this end, we compare rumor cascades where echo chamber members participate and the other rumor cascades in terms of structural (e.g., cascade size, depth, width) and temporal perspectives (e.g., propagation speed).

#### Cascade structure

**Figure 2:**
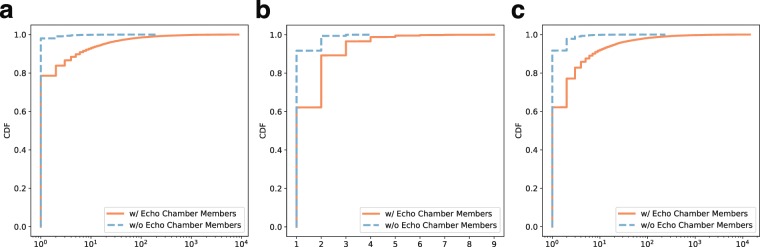
Structural properties of tweet rumor cascades. Rumor cascades where echo chamber members and non-members participate are compared in terms of size (**a**), height (**b**), and width (**c**). The rumor cascades with echo chamber members tend to be propagated to more audiences, deeper, and wider than the ones without members.

We first investigate the characteristics of the tweet rumor cascades, in which at least one echo chamber member participates, by comparing the cascades where no echo chamber member participates, in terms of size, height, and width of the cascade. As shown in Fig. 2, the rumor cascades with echo chamber members tend to be larger, deeper, and wider than those without echo chamber members. The size of 91.7% of the cascades without echo chamber members is only 1, which implies that rumor cascades without the participation by echo chamber members are unlikely to be propagated to others. On the other hand, the size, height, and width of the top 1% cascades with echo chamber members are higher than 247, 5, and 184, respectively, meaning that the cascades where echo chambers participate tend to be viral.

**Figure 3:**
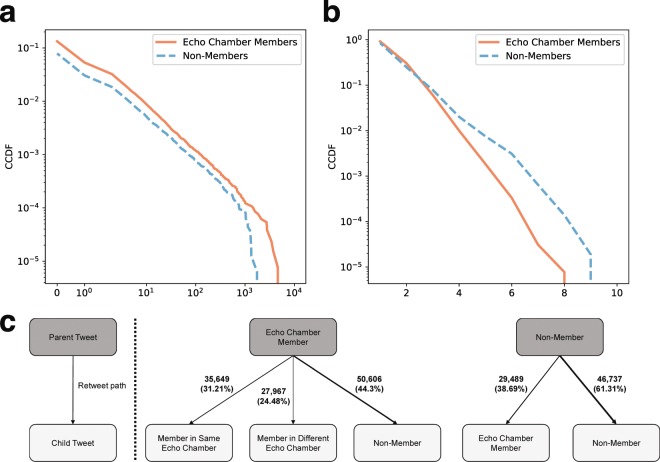
Roles of echo chambers in rumor cascades. Distributions of depth and number of retweets elicited by echo chamber members and by non-members are shown in (**a,b**), respectively. Most echo chamber members tend to be located at close to the roots of rumor cascades, and are likely to generate more retweets than non-members. The numbers (and portions) of retweets from members/non-members to members/non-members are described in (**c**). Overall, rumors tend more to be propagated to non-members while the portions of retweet paths from both members and non-members to non-members are different (44% and 61%, respectively). The portion of retweet paths between the members in the same echo chamber is higher than the ones in different echo chambers, meaning that rumors written by an echo chamber member tend to be more propagated among non-members or members in a same echo chamber.

To further examine the roles of echo chambers in rumor cascades, we compute (i) the depth of a tweet written by an echo chamber member and (ii) the number of retweets elicited by a tweet that an echo chamber member creates. Here, we only consider the cascades in which at least one echo chamber member participates. Figure 3 shows the distributions of depth and number of retweets elicited by the members (and non-members) of echo chambers. As shown in Fig. 3(a), the depths of the tweets by echo chamber members are lower than those of the non-members, meaning that the members are likely to participate in rumor cascades at the earlier stage than non-members. When we look at Fig. 3(b), echo chamber members tend to elicit more retweets. This implies that echo chamber members are likely to play more roles in propagating rumors by participating in earlier stages of the propagation and attracting more retweets, than non-members.

We next perform an in-depth analysis on how rumors are propagated among echo chamber members or non-members by counting the number of retweet paths, each of which consists of a parent and child tweets. In particular, we consider five cases: from an echo chamber member to (i) a member in the same echo chamber, (ii) a member in another echo chamber, and (iii) non-member, and from a non-member to (iv) a member in any echo chamber and (v) a non-member. Note that rumors may not be propagated by a pair of the members in the same echo chamber; a member of a rumor echo chamber may participate in different rumor cascades originated from a tweet written by either non-members or a member in a different echo chamber.

As shown in Fig. 3(c), rumors tend more to be propagated to non-members while the portions of retweet paths from both members and non-members to non-members are different (44% and 61%, respectively). In particular, the portion of retweet paths from non-members to non-members is much higher than the one from non-members to echo chamber members (61% and 39%, respectively), which means that rumors written by non-members are usually propagated not to echo chamber members but to user who are not involved in any echo chambers. Note that the portion of retweet paths between the members in the same echo chamber is higher than the ones in different echo chambers (31% and 24%, respectively), meaning that rumors written by an echo chamber member tend to be more propagated among non-members or members in a same echo chamber.

#### Propagation speed

**Figure 4:**
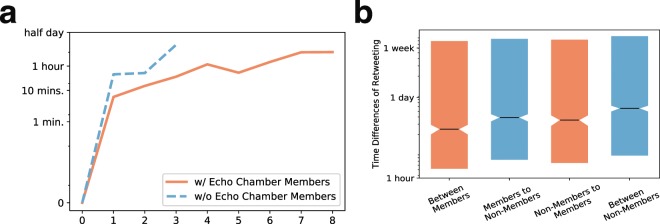
Speed of rumor propagation. We plot the depth increment times of rumor cascades with and without echo chamber members (**a**), and the distribution of the propagation times (**b**). Rumor cascades with echo chamber members are spread more quickly than those without members. The propagation times toward echo chamber members (orange boxes) are shorter than the ones in the case of retweet-paths toward non-members. In addition, the time differences from members are shorter than those from non-members. These results imply that echo chamber members contribute to propagate rumors quickly, by not only retweeting the rumor quickly but also eliciting other users’ quick responses.

To examine how echo chambers affect speed of rumor propagation, we measure the depth increment time of each rumor cascade. That is, for a given depth *i*, we plot the time difference between two (re)tweets that increase the depth of the cascade from depth *i* − 1 to *i*. Figure 4(a) shows the median values of the depth increment times for rumor cascades where members and non-members of echo chambers participate. Note that the depth increment times in the case of cascades with only non-members stop at 3 since the heights of all the cascades with only non-members are equal or less than 4. The depth increment time mostly increases as the depth increases, meaning that rumors tend to be propagated quickly in the early stages, but the propagation speed gets slower as the cascade becomes deeper. The depth increment times of rumor cascades with echo chamber members are substantially shorter than the ones without members, which implies that rumors in which echo chamber members are involved in tend to be spread quickly.

We next analyze the distributions of the propagation times for four types of retweet paths: (i) between echo chamber members, (ii) from an echo chamber member to a non-member, (iii) from a non-member to an echo chamber member, and (iv) between non-members. As shown in Fig. 4(b), the propagation times toward echo chamber members (orange boxes) are slightly shorter than the others. When we compare two blue boxes (i.e., propagation towards non-members), the time differences from echo chamber members are shorter than the ones from non-members. These results imply that echo chamber members are more likely to play roles in quick rumor spreads, by not only retweeting fast but also eliciting other users’ quick responses.

### Modeling and analyzing echo chamber network

#### Network analysis

**Figure 5:**
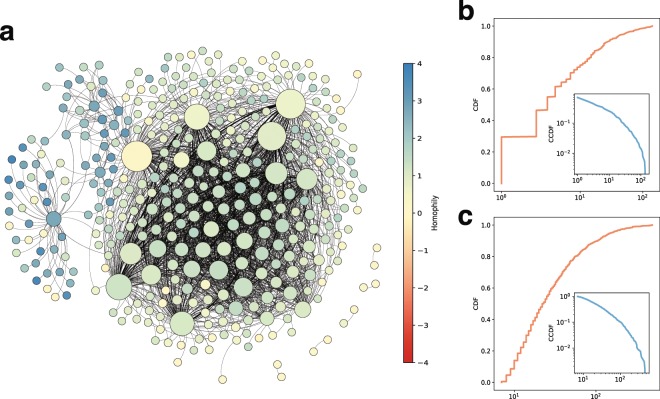
The echo chamber network. We depict the echo chamber network (**a**). The size and color of nodes indicates degree and member homophily, respectively. A node is close to blue or red if the homophily score is close to 4 or −4, respectively. The yellow nodes represent that the homophily score is close to 0. Distributions of degrees and weights of the echo chamber network are plotted at (**b**,**c**). Both distributions follow a heavy-tail distribution, which spans up to several orders of magnitudes.

We build and analyze the notion of an echo chamber network where nodes are a set of echo chambers and an edge represents a set of users involved in both echo chambers, depicted in Fig. 5(a). The size and color of a node reflects the degree and the homogeneity score of an echo chamber, respectively. That is, a color of a node is close to blue, yellow, or red when its homogeneity score is close to 4, 0, or −4, respectively. (The interactive version of the echo chamber network is available at https://daejin-choi.github.io/datasets/19srep-rumors/). As shown in Fig. 5(a), there exists a large and densely connected community that consists of the echo chambers whose homogeneity scores are around 0, meaning that members with diverse political standpoints are mixed in these echo chambers and actively participate in other echo chambers. When we look at the left side of the network, there are a few echo chambers whose sizes are relatively small and colors are close to blue, meaning that the members in these echo chambers tend to have similar political standpoints, and not to participate in other echo chambers. Note that these echo chambers are likely to be connected to other echo chambers that show strong positive homogeneities, which reveals that echo chambers whose members are politically skewed are likely to participate in other echo chambers that share politically skewed views.

Figure 5(b,c) show the distributions of degrees and weights of the given echo chamber network. As shown in Fig. 5(b), the degree distribution follows a power-law distribution that exhibits a heavy tail, which spans up to several orders of magnitudes. For instance, while 55% of nodes have less than 10 neighbors, the top 1% of nodes are connected to more than 227 echo chambers. The weight distribution also exhibits a heavy-tailed distribution. As shown in Fig. 5(c), the weights of around 78% edges are smaller than 10, and the weights of top 0.1% edges are greater than 301 (the maximum is 488). Such hub echo chambers with many connections (and/or high weights) to others can play important roles in propagating rumors by delivering rumors across each other.

#### Contributions by hub echo chambers in rumor propagation

**Figure 6:**
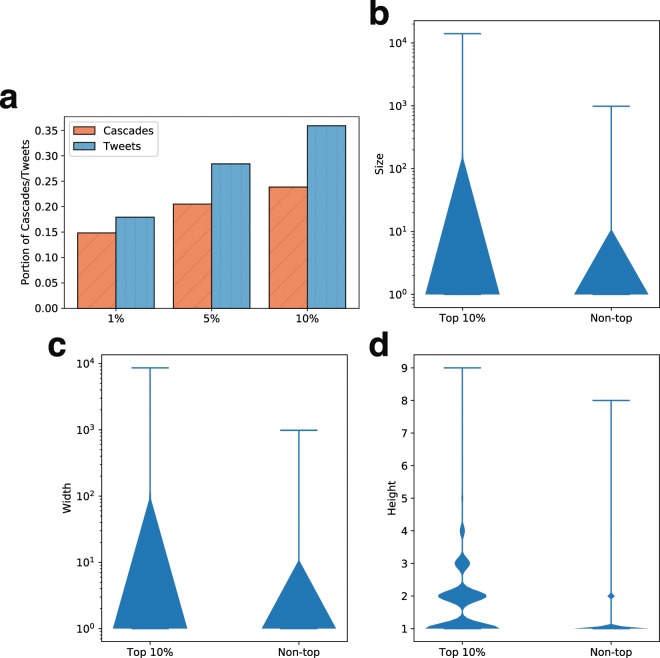
Contributions by hub echo chambers in rumor propagation. The top echo members in terms of degree tend to actively participate in rumor propagation and the rumor cascades where the members in these echo chambers are involved tend to be larger, wider, and deeper, which implies that participation from these members can be an signal to find viral rumor propagation.

We next explore how echo chambers, who play central roles in the network, contribute to and affect rumor propagation. To this end, we first choose the top 1%, 5%, and 10% echo chambers in terms of degree, and analyze the portions of cascades and tweets contributed by the top echo chambers, as described in Fig. 6(a). The top 1%, 5%, and 10% of nodes by degrees participate in 15%, 20%, and 24% of cascades by posting 18%, 28%, and 36% of tweets, respectively, meaning that the the members in these echo chambers tend to actively propagate rumors, i.e., writing more retweets across more rumors than others. Figure 6(b–d) show the distributions of the size, width, and height of rumor cascades where the top 10% echo chambers (by degrees) are involved. Here, we also plot the distributions of the other rumor cascades where none of users participate in the top 10% of echo chambers for a comparison purpose. We find that the rumor cascades associated with the hub echo chambers tend to be larger, wider, and deeper, which implies that a set of particular active echo chambers play crucial roles in viral rumor spreads.

## Discussion

In this section, we discuss academic implication of our findings and limitation of our work.

### How to identify rumor echo chambers

An echo chamber, known as a closed system or a group of users who share similar interest and actively spread information to others, has been identified based on the similarity of personal traits like political standpoint in the social science literature^18,19^. While such an approach usually requires to collect private individual information, which may not be easily obtained in practice, we propose an alternative method that identifies echo chambers based on users’ participatory behaviors in rumor propagation, which can be relatively easily obtained from social media. The rumor echo chambers identified in this paper represent two primary properties (i.e., selective exposure and homophily of members) of echo chambers reported in the previous literature, which implies that our proposed method for identifying rumor echo chambers can be alternatively and easily applied when user information are not available.

### Detection and prevention of rumor spreads with rumor echo chambers

As the growing importance of preventing or detecting rumor propagation in online platforms, there have been research effort in detecting (or preventing) propagation of rumors or misinformation, which mostly have focused on the characteristics of rumor content or individuals’ behaviors^7,9,10,13^. On the other hand, this paper modeled and analyzed how echo chamber members participate in rumor propagation, and revealed that echo chamber members are likely to participate in the early stage of rumor propagation and to elicit quick and many responses from others. In addition, our echo chamber network analysis showed that there are hub echo chambers who play significant role in rumor propagation. We believe that understanding of roles of echo chambers in rumor propagation can be used in rumor detection models proposed in the previous literature, as a cue for preventing rumor propagation in early stage. For example, the tweets that hub echo chamber members start to share can be monitored to prevent possible viral rumor spreads.

### Limitation and future work

Despite the implication described above, this work has a few limitations. First, the method of identifying echo chambers can be improved by adding other information such as age, gender, or political standpoint of an individual. Second, we notice that the methods and results of this paper should be carefully generalized to rumors about other topics such as history, science, health, or even non-rumors. Lastly, although echo chamber is reported as a factor that affects rumor spreads in their early stages, detecting and preventing rumor spreads using echo chamber observations are not covered in this paper, which will be investigated in the future work.

## Methods

### Data collection

**Figure 7:**
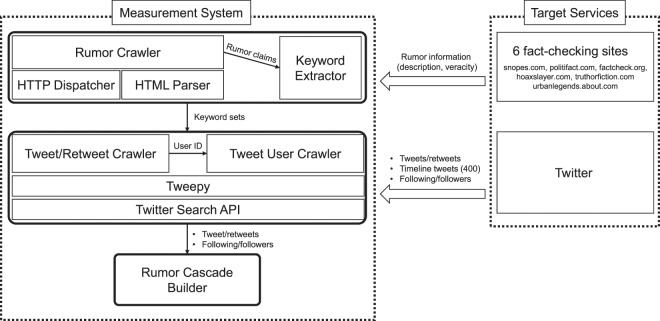
Rumor cascades measurement system. Our measurement system that crawls rumors, their associated tweets/retweets, and user information is depicted. We build each rumor cascade based on the collected tweets/retweets and their associated users’ following information.

To identify and analyze echo chambers in rumor propagation, we developed the data collection system as shown in Fig. 7, by which we crawled the rumors and their meta information from the six popular online debunking websites: snopes.com, politifact.com, factcheck.org, hoaxslayer.com, truthorfiction.com, and urbanlegends.about.com. Since all the above services did not provide APIs to collect data, we fetched every single web page and extracted rumor-related information including the claim (description of a rumor), veracity (e.g., true, false), category (e.g., politics, health), editor who determines the veracity of the rumor, and description of evidence. The description of rumors (i.e., claims) is delivered to the keyword extractor, which generates a set of words by tokenizing a given claim and removing stopwords. Note that we only considered the claims that include more than three words to avoid collecting trivial tweets/retweets. Finally, the generated set of words for a rumor is forwarded to the Twitter crawler in our system.

The Twitter crawler retrieved the tweets/retweets associated with rumors by using the Tweepy^23^, a Python library for accessing the Twitter Search API^24^. Among the collected tweets/retweets, we filtered out the retweets generated by bot accounts using the machine learning-based algorithm suggested in^25^. That is, the bot detection model accepts a user ID, gathers information of the user in Twitter, and builds more than 1,000 features including information diffusion patterns, social contacts of an account, inter-tweet time distribution, etc. Using the features and pre-trained model with high performance (AUC: 0.95), we finally identified 15.5% accounts as bots, and their tweets/retweets are removed while building rumor cascades. We also collected the information of users associated with the collected tweets/retweets, including the follower/following information and all the tweets posted on the timeline.

Using the collected tweet/retweets of the crawled rumors, we build *rumor cascades*, where nodes and edges represent a set of tweet/retweets and retweeting actions, respectively. Since the crawled retweets include only the original tweet information, we infer the retweet path of a tweet by using the original post ID and users’ follower information. That is, for a given original tweet *t* written by user *i*, we first extracted a set of retweets whose original tweet is *t* (denoted as *S*_*t*_). We then chose a subset of retweets whose elements (i.e., retweets) are written by *i*’s followers (denoted as *S*_*t*_) which are deemed child retweets of the original tweet. To find retweet paths of the retweets in *S*_*t*_, we first sorted the retweets in *S*_*t*_ in a chronological order and then inferred retweet paths of each retweet with a similar manner. Note that this approach assumes that users may tend to retweet from earlier tweet/retweets. By recursively computing retweet paths until all tweet/retweets in *S*_*t*_ found the path, we can finally build a retweet cascade that represents a rumor cascade. Note that we only consider rumor cascades whose sizes are equal to or greater than 100 in our analysis.

Collecting rumors and their associated rumor cascades from Jan. 2018 to Oct. 2018 for 10 months, we finally obtain 125 rumors whose veracity is true, false, or mixed, and 37,417 rumor cascades consisting of 289,202 tweets/retweets written by 176,362 users. Note that we keep the terms of use of all target services during the data collection process, and the collected dataset is anonymized.

### Definition of rumor echo chamber

**Figure 8:**
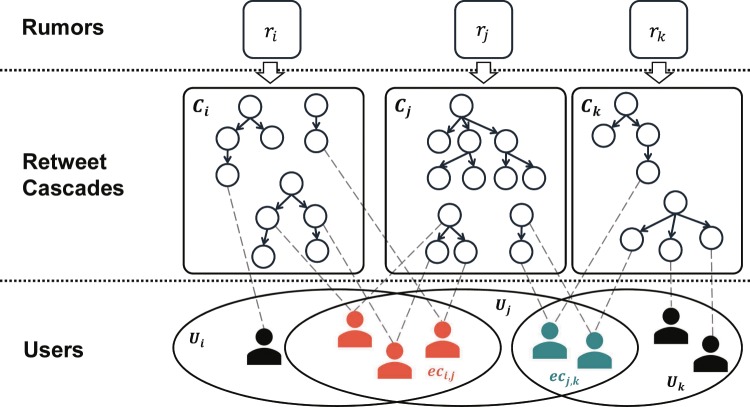
Identifying a rumor echo chamber. We first consider a set of the cascades (*C*_*i*_) for a given rumor *r*_*i*_, where the cascades are built from the tweets written by a set of users *U*_*i*_. We then compute a set of echo chambers *EC* whose element *ec*_*i,j*_ is the intersection of *U*_*i*_ and *U*_*j*_, corresponding a given pair of rumors *r*_*i*_ and *r*_*j*_, respectively.

The term ‘echo chamber’ generally refers to a closed system or a group of users who share similar interest and actively spread information to others, resulting in assimilation or even amplification of beliefs or memes^15^. Most of the prior work considered the echo chamber as a group of users who have similar political views^15,19,26^. However, identifying the political stance or orientation of individual user is often not easy since it is considered as a sensitive private information. In this paper, we define a *rumor echo chamber* as a group of users who have participated in spreading at least two common rumors. Here, we identify a rumor echo chamber based on users’ revealed activities, i.e., propagating common rumors, not based on users’ private information, e.g., political stance. To this end, we first consider a set of the cascades (*C*_*i*_) for a given rumor *r*_*i*_, where the cascades are built from the tweets written by a set of users *U*_*i*_. We then compute a set of echo chambers *EC* whose element *ec*_*i,j*_ is the intersection of *U*_*i*_ and *U*_*j*_, corresponding a given pair of rumors *r*_*i*_ and *r*_*j*_, respectively. Figure 8 illustrates how a rumor echo chamber is identified. Note that a user can be a member of multiple echo chambers, and we only consider echo chambers that each contains more than 2 users (i.e., |*ec*_*i,j*_| ≥ 2). We finally obtain 5,637 rumor echo chambers, and the number of users who belong to at least one rumor echo chamber is 36,185. The average number of members in identified echo chambers is 27.77. Note that, if we identify echo chambers with three or four common rumors, the average numbers of members in those echo chambers are 4.59 and 2.43, respectively, which may be too small to be analyzed. To avoid such a bias, we identify an echo chamber based on two common rumors.

### Modeling an echo chamber network

We model the relations among echo chambers by the notion of a *echo chamber network*, an undirected and weighted graph *G* = (*V*, *E*, *W*), where *V* is the set of echo chambers and *E* is the set of relations, where *e*_*ij*_ ∈ *E* is a set of users who participate in both of the chambers (*i* and *j*). Since a pair of echo chambers can share a common rumor, we only consider the edges where a pair of echo chambers are identified from disjoint rumors. The weight is calculated as the number of users in *e*_*ij*_, which is |*e*_*ij*_|. Since the echo chamber network is much dense (|*V*| and |*E*| are 3,622 and 496,328, respectively), we adopt a backbone extraction method^27^ to identify the statistically significant links in the given echo chamber network. By choosing *α* = 0.04, we finally build an echo chamber network that consists of 367 nodes and 2,005 edges.

## Data Availability

The information of the rumors investigated in this paper can be accessible at https://daejin-choi.github.io/datasets/19srep-rumors/. The other data analyzed during the current study is available from the corresponding author on the reasonable request.
